# Micrometer Giving without Calculation the Dimensions of Microscopic Objects

**Published:** 1860-02

**Authors:** 


### Selections.

Micrometer giving without Calculation the Dimensions of Microscopic Objects.—[We translate from Dr. Brown-Sequard's *Journal de Physiologie* the following note by Dr. Corelier, on a new micrometer.]

The measuring of objects is undeniably of the utmost importance for the microscopist. This is at present accomplished by means of an arbitrary scale, placed beneath the ocular. The relation of this scale to a millimeter divided into one hundred parts is first obtained; then ascertaining how many degrees of the scale are occupied by the object we wish to measure, we obtain the required dimensions by means of multiplication and division. This calculation, simple as it is, becomes tedious by repetition; it may be avoided in the following manner:—I place in the focus of the ocular a scale in which the millimeter is divided into ten parts such as is found in all modern microscopes. I then ascertain the proportion existing between this division and the millimeter divided into 100 parts, or micrometer. Suppose that 27 divisions of the ocular equal 19 hundreths of the millimeter; I have another scale made, in which 27 tenths of a millimeter are divided into 19 parts. This new scale placed beneath the ocular gives at once, and without calculation, the hundredths of a millimeter, as can be easily ascertained by looking, in the microscope, at the millimeter divided into 180 parts, for the division of the two scales will exactly correspond. To obtain at once the thousands it would only be necessary to divide the 27 tenths of a millimeter into 190 parts, &c. Of course it is necessary always to employ the same objective, and hence it is well to construct the scale for the one which we are most in the habit of using. It is now a long time since I have made these micrometers for my own use, and I have found them so convenient that I deem it my duty to explain their mode of construction, and to recommend their employment.—*Boston Med. and Surg. Jour.*

